# Adjunctive Use of Ketamine for Benzodiazepine-Resistant Severe Alcohol Withdrawal: a Retrospective Evaluation

**DOI:** 10.1007/s13181-018-0662-8

**Published:** 2018-05-10

**Authors:** Poorvi Shah, Marc McDowell, Reika Ebisu, Tabassum Hanif, Theodore Toerne

**Affiliations:** 1 Department of Pharmacy, Advocate Christ Medical Center, 4440 W. 95th Street, Room 022E, Oak Lawn, IL 60453 USA; 2 Department of Pharmacy, Minneapolis VA Health Care System, 1 Veterans Drive, Minneapolis, MN 55417 USA; 3 Department of Pulmonology, Advocate Christ Medical Center, 4440 95th St, Oak Lawn, IL 60453 USA; 4 Department of Emergency Medicine, Advocate Christ Medical Center, 4440 95th St., Oak Lawn, IL 60453 USA

**Keywords:** Alcohol withdrawal, Benzodiazepines, Ketamine, Delirium tremens

## Abstract

**Introduction:**

Benzodiazepine (BZD)-resistant alcohol withdrawal remains a challenge for most institutions due to limited evidence with available agents. One published study currently exists utilizing the *N*-methyl-d-aspartate antagonist, ketamine, for alcohol withdrawal.

**Objective:**

The purpose of our study was to evaluate the effect of adjunctive ketamine continuous infusion on symptom control and lorazepam infusion requirements for BZD-resistant alcohol withdrawal patients in the intensive care unit.

**Methods:**

A retrospective review was conducted of patients receiving ketamine adjunctively with a lorazepam infusion for severe alcohol withdrawal between August 2012 and August 2014. Outcomes included time to symptom control, lorazepam infusion requirements, ketamine initial and maximum daily infusion rates, and adverse effects of ketamine.

**Results:**

Thirty patients were included in the analysis. Mean time to initiation of ketamine after the initiation of a lorazepam infusion was 41.4 h. All patients achieved initial symptom control within 1 h of ketamine initiation. Median initial ketamine infusion rate was 0.75 mg/kg/h and the average maximum daily rate was 1.6 mg/kg/h. Significant decreases in lorazepam infusion rates from baseline were observed at 24 h (− 4 mg/h; *p* = 0.01) after ketamine initiation. No patients experienced documented CNS adverse effects. Two patients experienced hypertension and no patients experienced tachycardia related to ketamine.

**Conclusion:**

Adjunctive ketamine could provide symptom control for BZD-refractory patients and may potentially reduce lorazepam infusion requirements. Future studies to determine optimal dosing, timing of initiation, and place in therapy for BZD-resistant alcohol withdrawal are needed. The mechanism of action via the NMDA receptor with ketamine may provide benefit for BZD-resistant alcohol withdrawal.

## Introduction

The pathogenesis of alcohol withdrawal syndrome (AWS) primarily involves suppression of the inhibitory receptor, γ-aminobutyric acid (GABA), and overstimulation of the excitatory receptor, *N*-methyl-d-aspartate (NMDA) [1]. An increase in catecholamine activity is also observed during alcohol withdrawal due to inhibition of α_2_ receptors, further increasing sympathetic activity [2]. Clinical manifestations of alcohol withdrawal include tachycardia, hypertension, diaphoresis, agitation, tremor, hallucinations, seizures, and progression to delirium tremens (DTs), the most severe form of alcohol withdrawal [1–3]. Severe AWS is often managed in intensive care units (ICUs) and may require continuous sedative infusions, mechanical ventilation, and a prolonged ICU stay [2, 4].

Benzodiazepines (BZDs) have been considered first-line agents in the management of AWS due to their ability to mediate the GABA-A receptor and ameliorate physical and neurologic symptoms [3, 5]. BZDs have demonstrated the ability to prevent and treat seizures and DTs [1]. In certain patients, refractory symptoms can occur despite high doses of BZD. This is described as BZD-resistant AWS and can be attributed to GABA-A receptor saturation leading to no further improvement in symptoms. While there is no standard definition of BZD resistance, one criteria suggested is the requirement of greater than 40 mg of diazepam (or equivalent) within 1 h, which may necessitate adjunctive treatment options [6].

Alternative sedative agents and combination therapies have been studied in the treatment of AWS including phenobarbital, dexmedetomidine, and propofol. Aside from propofol, none of these agents provide strong antagonism for the NMDA receptor. Due to respiratory depression with propofol, most patients require mechanical ventilation which can increase ICU length of stay [2, 4].

Ketamine, an NMDA antagonist, is a sedative agent used in mechanically ventilated patients [7, 8]. It has been shown that high doses of ethanol competitively antagonize NMDA receptors and during withdrawal, the unopposed NMDA receptors lead to excitatory symptoms [9]. Therefore, NMDA antagonists mimic the effects of ethanol on the NMDA receptor at high doses and may be an alternative agent for the treatment for AWS, especially when agents with GABA agonistic activity such as BZD and/or barbiturates are not adequately controlling symptoms [2, 9]. Another advantage of ketamine is the low potential for respiratory depression, therefore providing another therapeutic option for non-intubated patients. Compared with dexmedetomidine, ketamine acts directly on a specific pathophysiologic step within the complex AWS pathway and could prove useful as an adjunctive therapy to BZD in non-intubated patients with severe AWS [2, 10]. Currently, only one observational study has evaluated the use ketamine in AWS [10]. The objective of our study was to evaluate the effect of adjunctive ketamine infusion on symptom control and lorazepam infusion dose changes for patients with BZD-resistant severe AWS.

## Methods

### Patient Population and Setting

Patients admitted to the Medical Intensive Cardiac Care Unit (MICCU) at Advocate Christ Medical Center receiving ketamine continuous infusions between August 2012 and August 2014 were evaluated. The hospital is a 749-bed tertiary care, level 1 trauma center with five adult ICUs and three ICU step-down units. Patients receiving ketamine in the MICCU during the study time frame were identified using an electric health record (Cerner Powerchart, Kansas City, MO). Subjects were included if ketamine was utilized for greater than 1 hour for AWS. Patients were excluded if ketamine was utilized for indications other than AWS, did not receive concomitant lorazepam infusion, or received propofol or dexmedetomidine during the ketamine infusion. The study was approved by the Advocate Health Care Investigational Review Board.

Severe AWS at our institution during the time frame of this study was defined by a Clinical Institute Withdrawal Assessment, Revised (CIWA-Ar) score of greater than 20. Patients were transferred to the MICCU from general floors or the emergency department if the CIWA-Ar score remained greater than or equal to 20 despite the non-ICU alcohol withdrawal protocol, which utilized a symptom-triggered intravenous (IV) lorazepam dosing regimen (cumulative dose of 8 mg lorazepam in 1 hour and 12 mg lorazepam within 2 hours; equivalent to 40 and 60 mg diazepam, respectively) [11]. When patients were admitted for severe AWS to the MICCU, they were converted to the ICU Severe Alcohol Withdrawal protocol, which utilized higher doses of lorazepam boluses (8 mg IV every 15 minutes for 3 doses; cumulative of 24 mg in less than 1 hour; equivalent to 120 mg diazepam), followed by phenobarbital 260 mg IV initial dose, then 130 mg IV every 15 minutes up to 8 doses (maximum of 1300 mg cumulative dose), and a lorazepam infusion if lorazepam and phenobarbital boluses were not adequately controlling symptoms. The final step in the protocol for patients not responding to BZD and barbiturates was intubation and propofol. Nurses titrated lorazepam infusions and provided lorazepam and phenobarbital boluses based on the above outlined steps and based on CIWA-Ar triggers outlined within the protocol. Ketamine was not part of the severe alcohol withdrawal protocol but was used adjunctively with a lorazepam infusion per ICU team discretion as an attempt to prevent intubation and the need for propofol in patients with refractory symptoms or in attempt to reduce lorazepam infusion requirements in patients already intubated. Although not protocolized, ketamine was initiated after a lorazepam infusion was inadequate in controlling symptoms and was typically weaned off prior to discontinuation of the lorazepam infusion. Standard hospital dosing and titration guidelines for sedation were used for ketamine with a starting dose of 0.5 mg/kg/h and a maximum dose during the study time frame of 4.5 mg/kg/h. Initial standard dosing could be adjusted per physician discretion. If patients were intubated, CIWA-Ar assessments were no longer utilized and the Motor Activity Assessment Scale (MAAS) was performed for sedation assessment. MAAS is a 7-point sedation score with a score of ≥ 4 indicating a restless, agitated, or combative patient. At the end of the study period in August 2014, the MAAS was converted to the Richmond-Agitation Sedation Scale (RASS) for sedation monitoring in all intubated patients; however, this conversion occurred after the inclusion of all study patients.

### Data Collection

The following variables were collected: lorazepam and ketamine requirements at 1, 4, 8, 24, and 48 hours post-ketamine initiation and clinical outcomes associated with the use of these agents including intubation rates, reason for intubation, time to initial symptom control, additional adjunctive agents used in the management of AWS, duration of all continuous infusions related to AWS management, ICU length of stay, and adverse effects of ketamine. Time to initial symptom control was defined as a CIWA-Ar score less than 20 or if intubated, a MAAS score less than 4 within 1 hour of ketamine initiation. Total duration of all continuous infusions refers to the onset of the lorazepam infusion and the end of the final infusion, which was mainly lorazepam as ketamine was typically the first infusion to be discontinued. Adverse effects of ketamine that were evaluated included documentation of central nervous system side effects including delirium, vivid imagery, or dream-like state, hypertension, and tachycardia. Hypertension was defined as a blood pressure increase within the first hour of ketamine initiation above 140/90 mmHg if previously below this threshold or a 10% increase above baseline if previously above 140/90 mmHg. Tachycardia was defined as a heart rate increase above 100 beats/min within 1 hour of ketamine initiation if previously below this threshold or a 10% increase above baseline if previously above 100 beats/min. Data was collected until ICU discharge or resolution of AWS if resolution occurred prior to discharge.

### Statistical Analysis

Data was analyzed using SAS 9.4 (Cary, NC, USA). Frequencies and percentages were utilized for categorical variables. Mean and standard deviations were used for normally distributed continuous variables and median and interquartile ranges used for skewed data. Two-group student’s *t* tests were conducted for comparison of lorazepam infusion doses from initial to follow-up time points with a *p* value of less than 0.05 as the threshold for significance.

## Results

### Baseline Characteristics

Baseline demographic data are presented in Table 1. Forty patients were screened based on intravenous ketamine infusions ordered during the study time frame, of which 30 patients met inclusion criteria. Patients enrolled were primarily men with an average age of 45 years presenting with a diagnosis of AWS. The primary symptom upon admission was agitation (56.7%). Approximately 23% of patients had a witnessed seizure and 30% of patients were tremulous upon admission. The main reason for ICU admission was alcohol withdrawal (90%). Immediately prior to initiating ketamine, the median CIWA-Ar score was 23 for non-intubated patients and the mean MAAS score was 4.6 for patients already intubated.

**Table 1:** Baseline demographics

	*n* = 30
Age, years: mean (SD)	45.6 (11.7)
Male: *n* (%)	26 (82.3)
Weight, kg: mean (SD)	90.4 (17.1)
Reason for ICU admission: *n* (%)	
Alcohol withdrawal	27 (90.0)
COPD exacerbation	1 (3.3)
Respiratory failure	2 (6.7)
Alcohol withdrawal symptoms upon admission: *n* (%)	
Agitation	17 (56.7)
Hallucination	6 (20.0)
Tremors	9 (30.0)
Obtunded	6 (20.0)
Seizure	7 (23.3)
Serum alcohol on admission, mg/dl: mean (SD)	155.4 (154.6)
Initial CIWA-Ar prior to initiation of ketamine: median (IQR)	23 (20–31)
Initial MAAS prior to initiation of ketamine: mean (SD)	4.6 (1.1)

*CIWA-Ar*, Clinical Institute Withdrawal Assessment, Revised; *COPD*, chronic obstructive pulmonary disease; *ICU*, intensive care unit; *IQR*, interquartile range; *MAAS*, Motor Activity Assessment Scale; *SD*, standard deviation

### Medication Dosing and Duration

Treatment data is presented in Table 2. Ketamine was initiated at variable doses during the study process. At the time of the study, our institution’s starting dose of ketamine was 0.5 mg/kg/h which could be titrated up to a maximum of 4.5 mg/kg/h for ICU sedation; however, the starting dose for alcohol withdrawal was determined at the discretion of the physician. The median initial dose of ketamine was 0.75 mg/kg/h and average maximum daily infusion dose was 1.6 mg/kg/h. Ketamine was initiated approximately 41 hours after a lorazepam infusion was started and the average lorazepam infusion rate was 14 mg/h at ketamine initiation. Phenobarbital was usually administered prior to ketamine (86.7%) as were the majority of lorazepam boluses; however, despite these therapies, patients remained dependent on moderately high doses of a lorazepam infusion with severe alcohol withdrawal demonstrated by CIWA-Ar scores above 20. Diazepam was the only adjunctive medication administered during the course of ketamine therapy in seven patients for the purpose of weaning from benzodiazepine infusions. The decision to utilize diazepam for this purpose was at the discretion of the ICU team and not part of the alcohol withdrawal protocol. The total duration of continuous infusions from the initiation of the first infusion to the cessation of the last was 109 hours, which also reflects the total lorazepam infusion duration as ketamine was the second infusion initiated and the first infusion discontinued. The duration of ketamine was approximately 54 hours.

**Table 2:** Treatment

	*n* = 30
Ketamine	
Time from lorazepam infusion initiation to ketamine initiation, hours: mean (SD)	41.4 (39.3)
Initial dose of ketamine, mg/kg/h: median (IQR)	0.75 (0.5–1.0)
Maximum daily infusion dose, mg/kg/h: mean (SD)	1.6 (0.9)
Total duration of ketamine infusion, hours: mean (SD)	53.7 (39.4)
Lorazepam	
Total lorazepam boluses prior to ketamine initiation, mg: mean (SD)	105.8 (79.1)
Total lorazepam boluses post-ketamine initiation, mg: median (IQR)	30 (0–66)
Lorazepam continuous infusion rate at time of ketamine initiation, mg/h: mean (SD)	14.3 (6.0)
Duration of lorazepam infusion post-ketamine cessation, hours: median (IQR)	20 (6–67)
Total duration of lorazepam infusion, hours: median (IQR)	109 (63–130)
Phenobarbital	
Patients receiving phenobarbital prior to ketamine initiation: *n* (%)	26 (86.7)
Total phenobarbital administered prior to ketamine initiation, mg: mean (SD)	972.3 (470.1)
Total phenobarbital administered post-ketamine initiation, mg: median (IQR)	0 (0–65)
Total duration of all infusions, hours: mean (SD)	109 (64.8)
Other adjunctive medications	
Diazepam: *n* (%)	7 (23.3)

*SD*, standard deviation; *IQR*, interquartile range

### Outcomes

**Fig. 1:**
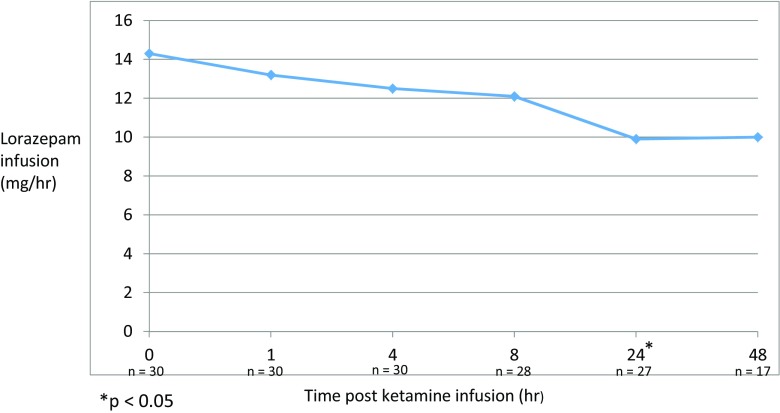
Average lorazepam infusion rate after ketamine initiation. Lorazepam infusion rates were averaged at 1, 4, 8, 24, and 48 hours after ketamine initiation for all patients with complete data available. The time at point 0 represents the initiation of ketamine

A reduction in lorazepam infusion rates was first observed beginning 1 hour after initiation of ketamine. A significant dose reduction was observed at 24 hours (*p* < 0.05; Fig. 1); however, whether this is due to the natural time course of resolution of symptoms or due to ketamine could not be determined due to the late initiation of ketamine. Within 48 hours of ketamine initiation, 13 patients (43%) were completely weaned off all infusions. Initial symptom control was obtained within 1 hour of ketamine initiation for all patients. The majority of patients were intubated (73.3%); however, most were intubated prior to ketamine therapy (72.7%). For the remaining six patients intubated after ketamine was initiated, the median duration of ketamine before intubation was approximately 8.5 hours. Reasons for intubation for these patients are listed in Table 3 with two patients intubated for escalation in symptoms despite ketamine. The average duration for mechanical ventilation for all patients was 5.4 days and the overall ICU length of stay was 8.2 days (Table 3). No patients experienced CNS adverse effects based on available documentation in the medical record. Hypertension and tachycardia secondary to ketamine occurred in two patients (6.7%) and no patients, respectively.

**Table 3:** Outcomes

	*n* = 30 (unless otherwise indicated)
Percent of patients achieving symptom control within one hour from ketamine initiation, hours: *n* (%)	30 (100)
Ventilator outcomes	
Intubated: *n* (%)	22 (73.3)
Intubated prior to ketamine: *n* (%), *n* = 22	16 (72.7)
Reason for intubation for all patients: *n* (%), *n* = 22	
Hypercapnic respiratory failure	10 (45.4)
Uncontrolled symptoms	8 (36.4)
Aspiration	4 (18.2)
Time from initiation of ketamine to intubation, hours: median (IQR), *n* = 6	8.5 (5–12)
Reason for intubation for patients currently on ketamine, *n* (%), *n* = 6	
Hypercapnic respiratory failure	2 (33.3)
Uncontrolled symptoms	2 (33.3)
Aspiration	2 (33.3)
Ventilator days: mean (SD)	5.4 (2.2)
ICU length of stay, days: mean (SD)	8.2 (2.4)
Adverse effects attributed to ketamine therapy	
CNS alterations: *n* (%)	0 (0)
Hypertension: *n* (%)	2 (6.7)
Tachycardia: *n* (%)	0 (0)

*IQR*, interquartile range; *SD*, standard deviation; *ICU*, intensive care unit

## Discussion

In this retrospective cohort study, patients were administered ketamine for the adjunctive management of AWS. Our results suggest that ketamine may reduce BZD requirements and provide symptom control. Ketamine was initiated relatively late in the treatment of AWS after significant amounts of BZD and phenobarbital had been administered. Ketamine dosing and monitoring were in accordance with our institution’s critical care infusion guidelines. However, the initial dosages of ketamine remained variable with an average rate of 0.75 mg/kg/h. This can be attributed to the infrequent use of ketamine as a sedative agent in the MICCU and differences in physician dosing preference. The ideal initial continuous infusion rate of ketamine for AWS is not yet clear due to the limited data currently available [4]. Post-ketamine initiation, all patients demonstrated initial control of symptoms within 1 hour when previous therapies were unsuccessful. However, two patients were subsequently intubated after ketamine initiation for further escalation in symptoms leading to additional complications. Of these two patients, one had subsequently increased CIWA-Ar scores which later decreased when the ketamine infusion was titrated upward to 1.5 mg/kg/h with concurrent lorazepam infusion at 10 mg/h and was intubated for concern of aspiration pneumonia. The second patient had subsequently increased CIWA-Ar scores with ketamine at 0.25 mg/kg/h and concurrent lorazepam infusion at 16 mg/h and was intubated due respiratory acidosis and increased secretions. The remaining four patients intubated after ketamine initiation maintained control of symptoms and causes of intubation included hypercapnic respiratory failure and aspiration likely due to complications of alcohol withdrawal. The late addition of ketamine after receiving large cumulative doses of BZD is another plausible explanation for the need for intubation.

Simultaneously, BZD requirements decreased after the addition of ketamine. Within 24 hours, average lorazepam infusion requirements were reduced by 28%. Within 48 hours, 43% of patients were completely weaned off all infusions. Due to the non-protocolized use of ketamine during the study, it is difficult to determine if patients remained on infusions due to continued symptoms or due to lack of downward titration per nurse discretion. Our hospital practice is to typically wean down and off ketamine first prior to weaning off lorazepam completely; however, since this was not part of a standardized protocol, these decisions were determined daily by the medical team. Using BZD reduction as an assessment of ketamine efficacy has been utilized in a previous study by Wong et al. [10]. Discriminating whether the BZD reduction in our population was due to the ketamine, phenobarbital or resolving AWS is difficult to determine, especially with late initiation of ketamine. In seven patients, scheduled enteral diazepam was initiated during the last hours of ketamine as a bridge for weaning off lorazepam continuous infusion. While this concomitant BZD may confound outcomes, diazepam was not introduced until after significant decreases in BZD requirements had been observed.

The majority of patients studied received ventilator support over their admission. Of the patients on ventilator support, approximately 68% were already intubated prior to receiving ketamine. The late addition of ketamine to therapy, approximately 41 hours after lorazepam infusion initiation, is a possible explanation for not only the increased BZD requirements prior to ketamine initiation but also the need for intubation. Earlier intervention with ketamine and earlier control of symptoms may have potentially avoided the need for aggressive airway support. Future studies focusing the administration of ketamine in non-intubated patients may expose benefits including decreased intubations and potentially decreased sedation requirements if AWS symptoms can be controlled earlier.

Safety data surrounding ketamine suffered from some limitations. The documentation of CNS adverse effects such as emergence reactions was difficult to obtain and if present, was difficult to differentiate from alcohol withdrawal delirium. Information regarding CNS adverse effects relied upon nursing documentation which poses its own limitations in a retrospective study. All our patients were on concurrent lorazepam infusions which could decrease the emergence reactions as has been reported with other BZD [12–14]. Cardiovascular effects such as tachycardia and hypertension are also known adverse effects of ketamine [15]. Within the first hour of ketamine initiation, only two patients experienced new onset or worsening of pre-existing hypertension and no patients experienced new onset or worsening of pre-existing tachycardia. Hemodynamic effects of ketamine are typically seen early within the first hour of administration which was the basis for our evaluation time frame [16, 17].

The only other published study utilizing ketamine for alcohol withdrawal in an ICU setting was conducted by Wong et al. [10]. Ketamine was utilized as an adjunctive agent for AWS. They evaluated sedation scores, diazepam equivalent requirements pre- and post-ketamine infusion for AWS, and duration of mechanical ventilation. The two studies have notable differences in methodology and results. In our study, all patients received a lorazepam continuous infusion compared with their study utilizing symptom-triggered benzodiazepine therapy. Approximately 83% of patients in the Wong study were BZD resistant. All our patients were BZD resistant since all patients required a lorazepam continuous infusion after failing multiple lorazepam boluses, which increases the potential need for mechanical ventilation. Previously, it has been reported that patients not responding to bolus therapy and requiring continuous sedative infusions may be more severely agitated with an increased length of stay and higher rate of respiratory failure requiring mechanical ventilation [2]. The dosing of ketamine utilized between our two studies also differed. Wong et al. reported a median infusion rate of 0.2 mg/kg/h compared with our initial rate of 0.75 mg/kg/h and average maximum daily rate of 1.6 mg/kg/h. These dosing differences could potentially explain the differences in outcomes related to symptom control and BZD reductions. With regard to outcomes, Wong et al. found no difference in alcohol withdrawal or sedation scores within 6 hours of ketamine initiation and demonstrated a non-significant trend in reduction of BZD use at 12- and 24-hours post-ketamine initiation. Our population demonstrated initial symptom control within 1 hour of ketamine initiation with CIWA-Ar scores decreasing below 20 that otherwise remained above 20 despite a lorazepam infusion rate of 14 mg/h. We also found a statistically significant reduction in lorazepam infusion rates at 24 hours. The duration of mechanical ventilation and ICU length of stays were shorter with Wong et al., which may potentially be explained based on different severities as the patients in our study all required continuous lorazepam infusions and could not be controlled with symptom-triggered lorazepam boluses. One plausible explanation for the differences in alcohol withdrawal scores and benzodiazepine reduction seen in our population may be due to the higher dosing strategy. At the time of the study, our institution’s sedation guidelines for ketamine as a sedative infusion allowed a maximum dose of 4.5 mg/kg/h, which explains the higher ketamine average maximum dose of 1.6 mg/kg/h. The pain, agitation, and delirium guidelines provide a dosing recommendation of 0.05–0.4 mg/kg/h for ketamine when used for routine ICU sedation [7]. Sedation requirements in AWS are often higher than the routine sedative infusion doses as has been reported with other agents [18–23]. Ketamine has been used for other indications such as status epilepticus in dosing ranges similar to our study [24]. The dosing of ketamine for alcohol withdrawal remains unknown and therefore extrapolating higher dosing used for other indications may be warranted for severe alcohol withdrawal patients with BZD resistance.

## Limitations

Limitations of the study include the retrospective design and lack of a control arm; therefore, the impact on clinical outcomes such as intubation rates, ventilator days, and ICU length of stay cannot be determined. The lack of a control arm has been a limitation in prior initial studies with ketamine and other adjunctive medications in alcohol withdrawal [10, 25–28]. Despite this limitation, our results provide insight into the potential need for higher ketamine dosing requirements to achieve symptom control and to decrease BZD requirements. The study was also limited due to the small sample size. Additionally, outcomes data that relied upon documentation from the medical team could be underreported. Ketamine was used at the discretion of the medical team and not currently part of a standardized protocol so the timing of initiation, dosing, and the use of adjunctive medications such as phenobarbital may have also contributed to the reduction in BZD. Most of our patients were already intubated prior to initiation of ketamine so the role of ketamine in preventing intubation if used earlier in the course of therapy remains to be determined.

## Conclusion

Our retrospective study evaluating the effect of ketamine as adjunctive treatment for severe AWS suggests that the addition of ketamine to a BZD infusion could enhance symptom control, decrease BZD infusion requirements, and may be tolerated with minimal adverse effects. Ketamine’s future role in AWS therapy requires additional studies evaluating the optimal dosage range, timing of initiation of ketamine, and the impact on intubation rates, ventilator days, and ICU length of stay. Its direct effect on the pathogenesis of alcohol withdrawal via an alternative mechanism may provide benefit as an adjunctive agent in BZD-resistant AWS in the ICU setting.

### Sources of Funding

None
